# Adaptive evolution of tembusu virus enhances mammalian pathogenicity and vector competence

**DOI:** 10.1128/spectrum.01094-26

**Published:** 2026-07-29

**Authors:** Tao Wang, Fan Liu, Yuyan Fan, Wei Li, Yu He, Zhen Wu, Rui Luo, Mingshu Wang, Renyong Jia, Anchun Cheng, Shun Chen

**Affiliations:** 1 Institute of Veterinary Medicine and Immunology, Sichuan Agricultural University 670358, Chengdu, Sichuan, China; 2 Research Center of Avian Disease, College of Veterinary Medicine, Sichuan Agricultural University 670358, Chengdu, Sichuan, China; 3 Agricultural Animal Diseases and Veterinary Public Health Key Laboratory of Sichuan Province, Sichuan Agricultural University, Chengdu, Sichuan, China; 4 Engineering Research Center of Southwest Animal Disease Prevention and Control Technology for Ministry of Education, Sichuan Agricultural University, Chengdu, Sichuan, China; 5 Key Laboratory of Agricultural Bioinformatics, Ministry of Education, Sichuan Agricultural University, Chengdu, Sichuan, China; 6 State Key Laboratory of Agricultural Microbiology, College of Veterinary Medicine, Huazhong Agricultural University, Wuhan, China; 7 Veterinary Department in College of Animal Science, State Key Laboratory of Green Pesticide, Institute of Veterinary Immunology and Green Drugs, Guizhou University, Guiyang, China; National University of Singapore, Singapore, Singapore

**Keywords:** Tembusu virus, pathogenicity, vector competence, mouse, mosquito

## Abstract

Tembusu virus (TMUV) is an avian orthoflavivirus responsible for severe egg-drop syndrome, inflicting substantial economic losses on the poultry industry in China and Southeast Asia. TMUV exhibits zoonotic potential and has diversified into distinct phylogenetic clusters; however, the phenotypic consequences of this genetic divergence remain poorly characterized. This study systematically evaluated the mammalian pathogenicity in mice and the vector competence in *Culex quinquefasciatus* mosquitoes of five TMUV isolates representing major clusters (clusters 2.1.1, 2.1.2, 2.2, 3.1, and 3.2). Results showed that contemporary duck-derived isolates (clusters 2 and 3.2) demonstrated superior replication efficiency and infection rates in mosquitoes compared to the ancestral mosquito-derived isolate (MM 1775, cluster 3.1); the recently emerged chicken-derived cluster 3.2 isolate (WH2025) caused 100% mortality in the infected mice, exhibiting the highest pathogenicity. These findings indicate that TMUV evolution has selected for phenotypes with enhanced mammalian virulence and increased its fitness in mosquito vectors, highlighting a growing risk of cross-species transmission. This study provides crucial insights for risk assessment and targeted surveillance for TMUV.

**IMPORTANCE:**

Tembusu virus (TMUV) is an emerging mosquito-borne flavivirus that has been circulating in China and Southeast Asia in recent years, causing significant economic losses to the waterfowl farming industry. In recent years, strains of TMUV cluster 3.2 have been increasingly isolated from laying hens and geese, and it has been reported that TMUV can cause mortality in mammals (dolphins). TMUV exhibits mosquito-borne transmission and potential zoonotic characteristics; however, there is a lack of systematic understanding regarding the vector transmission efficiency and changes in mammalian pathogenicity among different evolutionary clusters of TMUV. This study compares the adaptability to *Culex quinquefasciatus* and pathogenicity in mice of major TMUV evolutionary clusters, contributing to our understanding of TMUV’s mosquito-borne transmission capacity and mammalian pathogenicity. The findings are of great significance in assessing the transmission capacity and strain risks of epidemic TMUV strains.

## INTRODUCTION

In 2010, an emerging Tembusu virus (TMUV) associated with a severe egg-drop syndrome caused outbreaks in southeastern China. Since then, TMUV has become endemic in China and Southeast Asia, causing substantial economic losses in the duck industry (1, 2). TMUV belongs to the genus *Orthoflavivirus* (formerly *Flavivirus*) within the family *Flaviviridae*, which also includes several medically important zoonotic pathogens such as dengue virus (DENV), Zika virus (ZIKV), and Japanese encephalitis virus (JEV) (3).

The first TMUV isolate (MM 1775) was isolated from *Culex tritaeniorhynchus* mosquitoes in Malaysia in 1955 and remained primarily associated with mosquitoes for decades (4). The scenario changed dramatically in 2010 with its emergence as an avian pathogen (1). It is now known to infect a broad spectrum of avian hosts, including ducks, chickens, geese, pigeons, sparrows, and quail, as well as multiple mosquito species (5, 6). Notably, TMUV has demonstrated zoonotic potential, evidenced by its identification in captive bottlenose dolphins (*Tursiops truncatus*) in Thailand, causing neurological disease and mortality (7), and by serological evidence of exposure among duck-farm workers (8–10).

Phylogenetic analyses of full-length TMUV genomes have classified circulating isolates into three major clusters: clusters 1, 2, and 3. In mainland China, cluster 2 is the predominant lineage and can be further subdivided into subclusters 2.1 and 2.2 (11). However, the functional consequences of this genetic diversity remain poorly understood. Since 2019, cluster 3.2, which includes an increasing number of chicken- and goose-derived isolates, has become prominent. Notably, cluster 3.2 isolates exhibit higher pathogenicity in chickens but lower pathogenicity in ducks compared to cluster 2 isolates (12, 13). This stark contrast suggests that distinct TMUV lineages may have evolved differential adaptation to specific hosts. Previous pathogenicity studies have typically focused on single isolates or limited comparisons within one cluster. A systematic, cross-cluster evaluation of TMUV isolates in parallel, using standardized models, is critical to unravel the relationship between viral phylogeny and pathogenic outcomes.

Similar to JEV and West Nile virus (WNV), TMUV is believed to be primarily transmitted by *Culex* mosquitoes. In previous study, the TMUV from the Cluster 1 had a higher viral load in the salivary glands and saliva after infecting the *Culex tritaeniorhynchus* mosquito compared to the TMUV strain from the Cluster 2.1 (14). However, a systematic evaluation of the vector competence of isolates across different phylogenetic clusters is still lacking.

Given that TMUV isolates from different clusters display variable pathogenicity in avian and mammalian hosts and potentially differ in their mosquito transmission efficiency (15), a systematic comparative assessment using representative isolates is warranted. In this study, we employed five TMUV isolates representing multiple phylogenetic clusters (clusters 2.1.1, 2.1.2, 2.2, 3.1, and 3.2) to infect mice and *Culex quinquefasciatus* mosquitoes. Our objective was to comprehensively assess their relative pathogenicity in a mammalian model and their vector competence in *Culex quinquefasciatus* mosquitoes. This study provides an integrated assessment of the phenotypic diversity underlying TMUV evolution, with critical implications for outbreak preparedness, surveillance, and understanding the determinants of flavivirus emergence.

## MATERIALS AND METHODS

### Cells and viruses

Baby hamster kidney (BHK-21) and African green monkey kidney (Vero) cells were maintained in Dulbecco’s modified Eagle medium (DMEM) supplemented with 10% fetal bovine serum (FBS) at 37°C in a 5% CO_2_ atmosphere. *Aedes aegypti* (Aag2) cells were cultured in Schneider’s Drosophila medium supplemented with 10% FBS at 28°C without CO_2_. The following TMUV isolates were propagated in Vero cells and titrated on BHK-21 cells: CQW1 (cluster 2.1.1, duck origin), CHN-YC (cluster 2.1.2, duck origin), MC (cluster 2.2, duck origin), MM1775 (cluster 3.1, mosquito origin), and WH2025 (cluster 3.2, chicken origin).

### Viral growth kinetics and plaque characteristics *in vitro*

Vero and Aag2 cells were seeded into 24-well plates and cultured overnight. The following day, cells were infected with 500 (Vero) or 5,000 (Aag2) TCID₅₀ of TMUV per well, respectively. After adsorption for 1 h, the inoculum was removed, cells were washed thrice with PBS and maintained in DMEM or Schneider’s medium containing 2% FBS. Culture supernatants were harvested at 12, 24, 48, 72, and 96 h post-infection (hpi). Viral titers in the supernatants were determined by endpoint dilution assay on BHK-21 cells and calculated using the Kärber method. Plaque assays were performed on confluent BHK-21 cell monolayers in 12-well plates. Serially diluted virus samples were adsorbed for 1.5 h at 37°C with gentle shaking every 15 min. Cells were then overlaid with DMEM containing 1.5% methylcellulose and 2% FBS. After 5–7 days of incubation, plaques were visualized by fixing cells with 4% paraformaldehyde and staining with 1% crystal violet.

### Mosquito vector competence studies

*Culex quinquefasciatus* mosquitoes were reared under standard insectary conditions (26°C ± 2°C, 70% relative humidity, with a 12:12 h light:dark cycle). To bypass the midgut barrier and directly assess viral dissemination and transmission potential, 5- to 7-day-old female mosquitoes were intrathoracically inoculated with 300 nL of TMUV (10^3^ TCID_50_/mL) using a nano-injector (RWD, China). To evaluate the complete infection cycle, including midgut infection and escape, mosquitoes were orally fed a virus-blood mixture (1:1 ratio of viral suspension and defibrinated sheep blood) containing 10⁵ TCID₅₀/mL of TMUV using a membrane feeding system (Hemotek, UK). Fully engorged females were selected and maintained. Mosquitoes were sampled at 3, 7, and 14 days post-inoculation (dpi), and viral loads were quantified by RT-qPCR.

### Pathogenicity assessment in mouse models

Three-week-old female Kunming mice were randomly divided into six groups (*n* = 14), and each one was infected with 30 μL of TMUV (10^4^ TCID_50_) via intracerebral (i.c.) injection, and the mock group was injected with an equal volume of DMEM. Eight mice per group (MM 1775 strain injection group included 5 mice) were monitored daily for body weight, signs of illness, and mortality. The clinical symptom scoring for mice referred to previous studies (16), including depression and sluggishness, paralysis, blindness, and death. Each mouse was assigned one point for each symptom observed. The total score was calculated as follows: (item 1/total number of mice per group) × 10 + (item 2/total number of mice per group) × 30 + (item 3/total number of mice per group) × 15 + (item 4/total number of mice per group) × 45. Mice exhibiting body weight loss of more than 20% or complete hind leg paralysis were euthanized. At 3 and 5 dpi, mice (*n* = 3 per group per time point) were euthanized to collect samples (i.e., brain, heart, liver, spleen, lung, and kidney) for viral load determination and histological examination. A portion of the mouse tissue was stored at −80°C for virus titration or RNA extraction, and another portion was fixed in 10% neutral formalin for histopathological processing. Mouse brain samples were fixed in buffered 10% formalin for 24 h, then dehydrated with graded alcohol, embedded in paraffin, and cut into 5-micron-thick sections. The sections were stained with hematoxylin and eosin by conventional methods.

### RNA isolation and RT-qPCR

Total RNA from mosquito and mouse tissues was extracted using RNAiso Plus (Takara), following the manufacturer’s protocols. RNA was reverse transcribed, and TMUV RNA copies were quantified by absolute RT-qPCR; the mRNA expression levels were quantified by relative RT-qPCR. All quantitative primers used in this study have been reported in previous studies and are listed in Table 1 (15, 16).

**TABLE 1:** The primer sequences used in this study for qPCR

Primer	Sequence (5'→3')	Target	Reference
DTMUV-qF	AAACCAGGAAGACCCG	TMUV CQW1, CHN-YC, MC, and WH2025 strains	15
DTMUV-qR	TGAAGAAAGTCAGTAGAGC		
TMUV-qF	GAAATCGAATCTGCCAGGAC	TMUV MM 1775 strain	16
TMUV-qR	CCGCTCACCCAATACATC		
qM-GAPDH-F	GATGGACACATTGGGGTT	Mouse GAPDH	
qM-GAPDH-R	AAAGCTGTGGCGTGATG		
qM-IFNβ-F	TTACACTGCCTTTGCCATCC	Mouse IFNβ	
qM-IFNβ-R	ACTGTCTGCTGGTGGAGTTCAT		
qM-IFNγ-F	ACTGGCAAAAGGATGGTG	Mouse IFNγ	
qM-IFNγ-R	GTTGCTGATGGCCTGATT		
qM-IL10-F	GCCCTTTGCTATGGTGTC	Mouse IL10	
qM-IL10-R	TCTCCCTGGTTTCTCTTCC		
qM-IL6-F	GTATGAACAACGATGATGCAC	Mouse IL6	
qM-IL6-R	CTCCAGAAGACCAGAGGAAA		
qM-IL1β-F	AGTTGACGGACCCCAAA	Mouse IL1β	
qM-IL1β-R	TCTTGTTGATGTGCTGCTG		
qM-Mmp3-F	GCTCATCCTACCCATTGC	Mouse Mmp3	
qM-Mmp3-R	GCTCCATACCAGCATCATC		
qM-TNFα-F	CGCTGAGGTCAATCTGC	Mouse TNFα	
qM-TNFα-R	GGCTGGGTAGAGAATGGA		
qM-IFNα-F	AATGCAACCCTCCTAGACTC	Mouse IFNα	
qM-IFNα-R	GGCTCTCCAGACTTCTGCTC		

### Phylogenetic analysis

We obtained all the available TMUV genome sequences isolated from 1955 to 2025 from the NCBI GenBank database (https://www.ncbi.nlm.nih.gov) until December 2025. The sequences of the virus isolates that were continuously passaged in cells were excluded. The sequence information of all of the TMUV isolates analyzed in this study can be found in Table S1. The nucleotide sequences were aligned by MAFFT v7.037b (17) and refined via MEGA v7.0 (18). The phylogenetic tree was generated using the maximum likelihood method implemented on MEGA v7.0 with default parameters, and the trees were visualized and annotated using ggtree (19).

### Statistical analysis

All data were analyzed using GraphPad Prism 9.0. Statistical analysis of the viral growth curves *in vitro* was performed using two-way ANOVA method followed by Tukey’s honestly significant difference post-hoc test. Statistical analysis of tissue viral loads, viremia, and cytokine expression levels in mice and *Culex quinquefasciatus* mosquitoes inoculated with different viral strains was performed using one-way ANOVA followed by Tukey’s multiple comparisons test. An adjusted *P* value of less than 0.05 was considered statistically significant.

## RESULTS

### Phylogenetic and distribution analysis of TMUV

**Fig 1:**
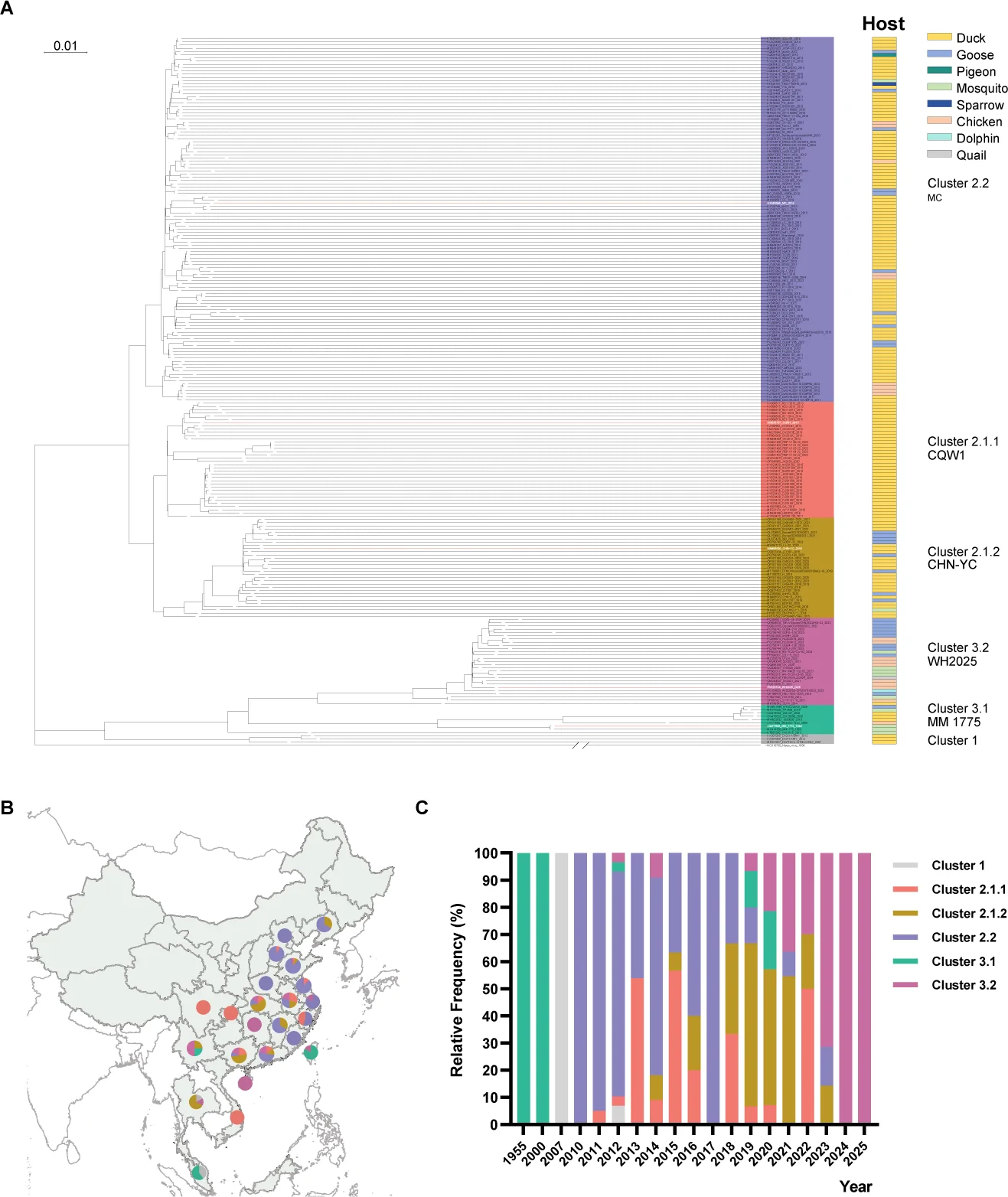
Analysis of TMUV evolution and spatio-temporal distribution. (**A**) Phylogenetic analysis of the TMUV genome sequences. (**B**) Geographical distribution of different TMUV lineages across global regions. (**C**) Temporal dynamics of TMUV lineages from 1955 to 2025. The Y-axis represents the percentage of lineages detected each year.

We performed phylogenetic analysis on all publicly available TMUV complete genome sequences (*n* = 219) from the NCBI database as of December 2025. TMUV was primarily divided into three major evolutionary lineages: clusters 1, 2, and 3. Cluster 1 only contained three isolates, and no more isolates were isolated from this cluster afterward. Cluster 2 was further subdivided into clusters 2.1 and 2.2, and 2.1 could be further differentiated into two subclusters: 2.1.1 and 2.1.2. The MM 1775 strain, isolated from mosquitoes, and some similar isolates formed cluster 3.1 on their own, while isolates obtained from chickens and geese in recent years aggregated to cluster 3.2 (Fig. 1A). Geographic distribution analysis revealed that the predominant isolates in mainland China belonged to clusters 2 and 3.2. In contrast, viruses within cluster 3.1 have been isolated only in Southeast Asia and Taiwan district (Fig. 1B). Notably, since 2019, the prevalent isolates circulating in mainland China have been primarily clusters 2.1.2 and 3.2 (Fig. 1C).

### Characteristics of TMUV isolates in mammalian and mosquito cells

**Fig 2:**
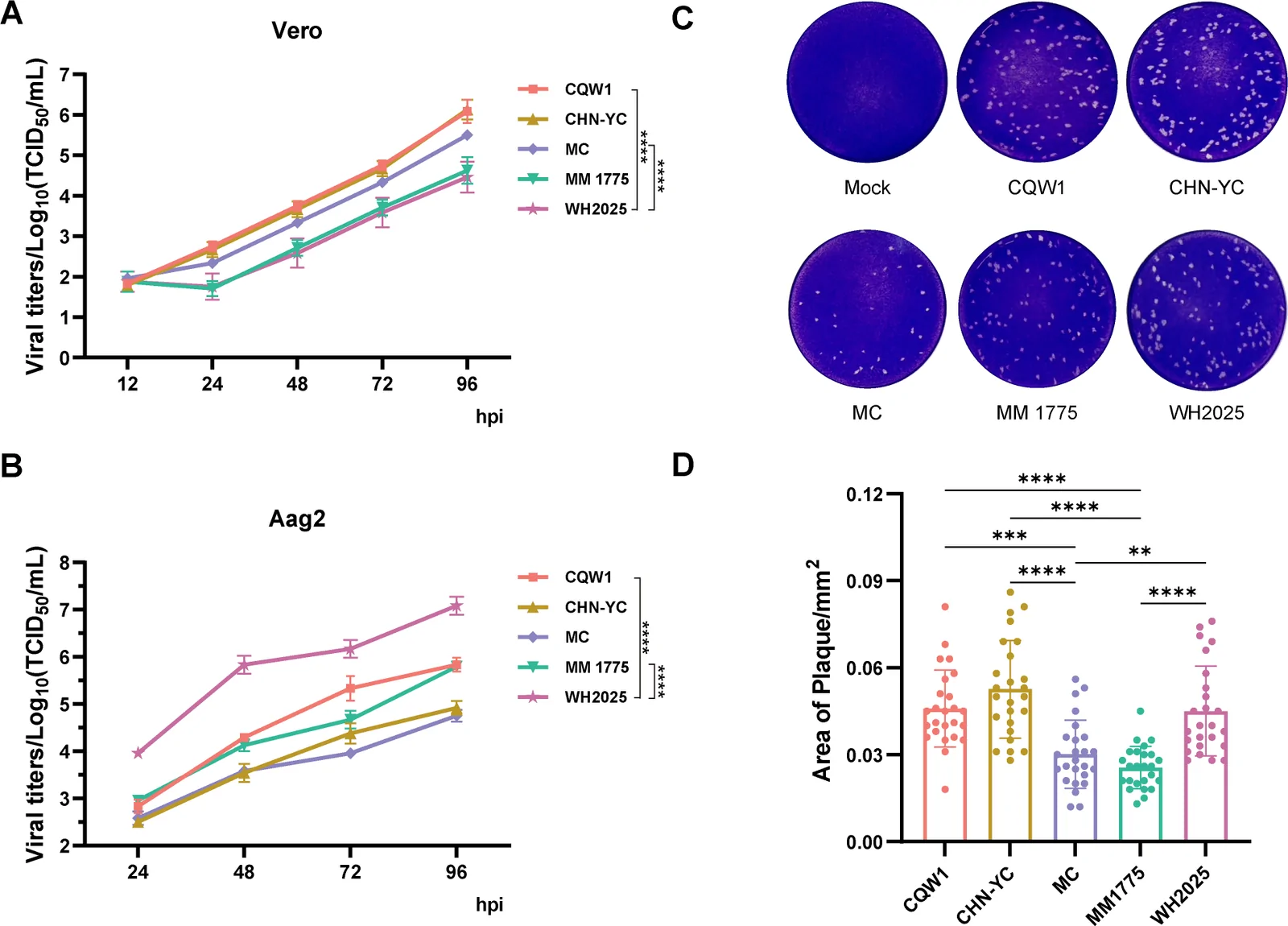
Characteristics of different TMUV isolates *in vitro*. (**A** and **B**) Vero cells (**A**) and Aag2 cells (**B**) were infected with TMUV isolates, and viral titers in the supernatant were determined using BHK-21 cells. (**C**) Plaque morphology of TMUV isolates analyzed on BHK-21 cells. (**D**) Areas of 25 randomly selected plaques per isolate were measured. Data are presented as means ± SD; a one-way ANOVA was used to assess the statistical significance of plaque size differences, and a two-way ANOVA was used for the viral growth curve analysis. *P < 0.05, **P < 0.01, ***P < 0.001, and ****P < 0.0001.

Vero and Aag2 cells were used to investigate the growth kinetics of different TMUV isolates. In Vero cells, strains CQW1 (cluster 2.1.1) and CHN-YC (cluster 2.1.2) replicated to the highest titers, whereas MM1775 (cluster 3.1) and WH2025 (cluster 3.2) exhibited the lowest replication efficiency (Fig. 2A). In Aag2 cells, WH2025 strain showed higher proliferation levels than other isolates; the CQW1 and MM 1775 strains had similar and middle growth levels, although the proliferation rate of CHN-YC isolates is fastest in Vero cells, CHN-YC and MC isolates were significantly lower than other isolates of TMUV (Fig. 2B). Although the proliferation efficiency of MC in Vero cells was close to that of CQW1 and CHN-YC, the size of the plaques it formed was significantly smaller than that of all other strains (Fig. 2C and D).

### Vector competence of different TMUV isolates

**Fig 3:**
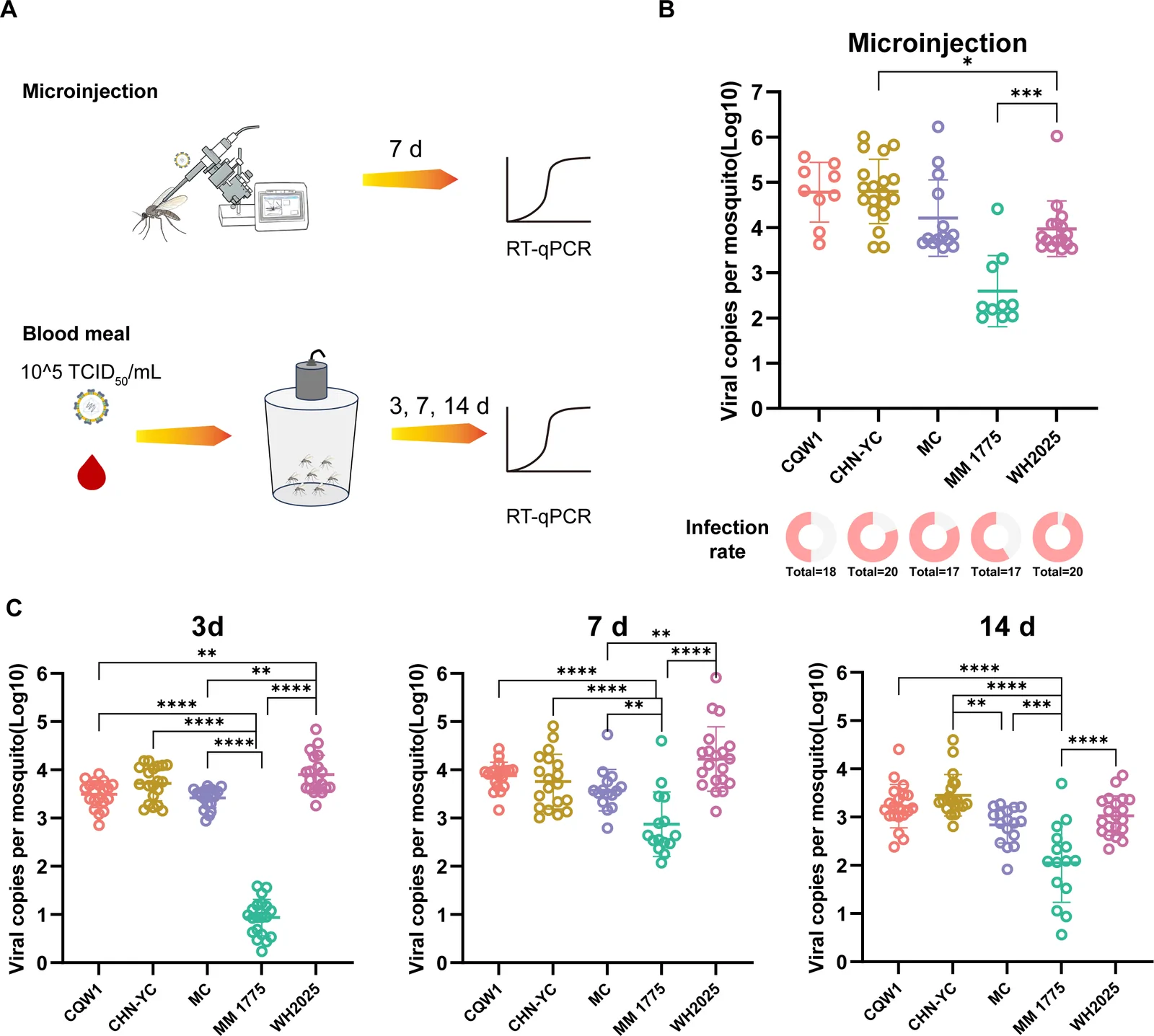
Vector competence of *Culex quinquefasciatus* for different TMUV isolates. (**A**) Experimental design for vector competence for TMUV. Female mosquitoes were infected with five TMUV strains via microinjection (upper panel) or blood meal (lower panel), and viral copies were detected at 7 dpi using RT-qPCR. (**B**) Viral genome copies in mosquitoes, which were infected via microinjection; the infection rate is defined as the number of infected mosquitoes divided by the total number of mosquitoes. (**C**) Viral genome copies in mosquitoes, which were infected via a blood meal. The statistical significance was determined by the one-way ANOVA method. *P < 0.05, **P < 0.01, ***P < 0.001, and ****P < 0.0001.

To evaluate the vector competence of TMUV isolates from different clusters, *Culex quinquefasciatus* mosquitoes were inoculated with TMUV via thoracic microinjection. Viral genome copies in whole mosquitoes were then quantified at 7 days post-inoculation, or at 3, 7, and 14 days post-blood meal (Fig. 3A). At a dose of 10^3.5^ TCID₅₀/mL, the WH2025 strain exhibited the highest infection rate (95%) among all tested isolates. Notably, the mosquito-derived isolate MM 1775 displayed both lower infection rates and viral copy numbers than the avian-derived TMUV isolates (Fig. 3B). To further assess vector competence under more natural conditions, mosquitoes were allowed to feed on blood meals containing TMUV. Viral genome copy numbers in mosquitoes were measured at 3, 7, and 14 days post-feeding (Fig. 3A). All TMUV isolates reached peak viral RNA levels by 7 days post-blood meal, followed by a decline in both viral titers and infection rates at 14 days post-blood meal. At this time point, MM 1775 exhibited an infection rate comparable to that of WH2025, but its viral RNA load was approximately ten-fold lower. Both CHN-YC and CQW1 maintained higher infection rates and viral loads than MM 1775 at this time point (Fig. 3C). The infection ability of different TMUV isolates against *Culex quinquefasciatus* and the proliferation level of the virus in mosquitoes show significant differences.

### Virulence of different TMUV isolates for mice

**Fig 4:**
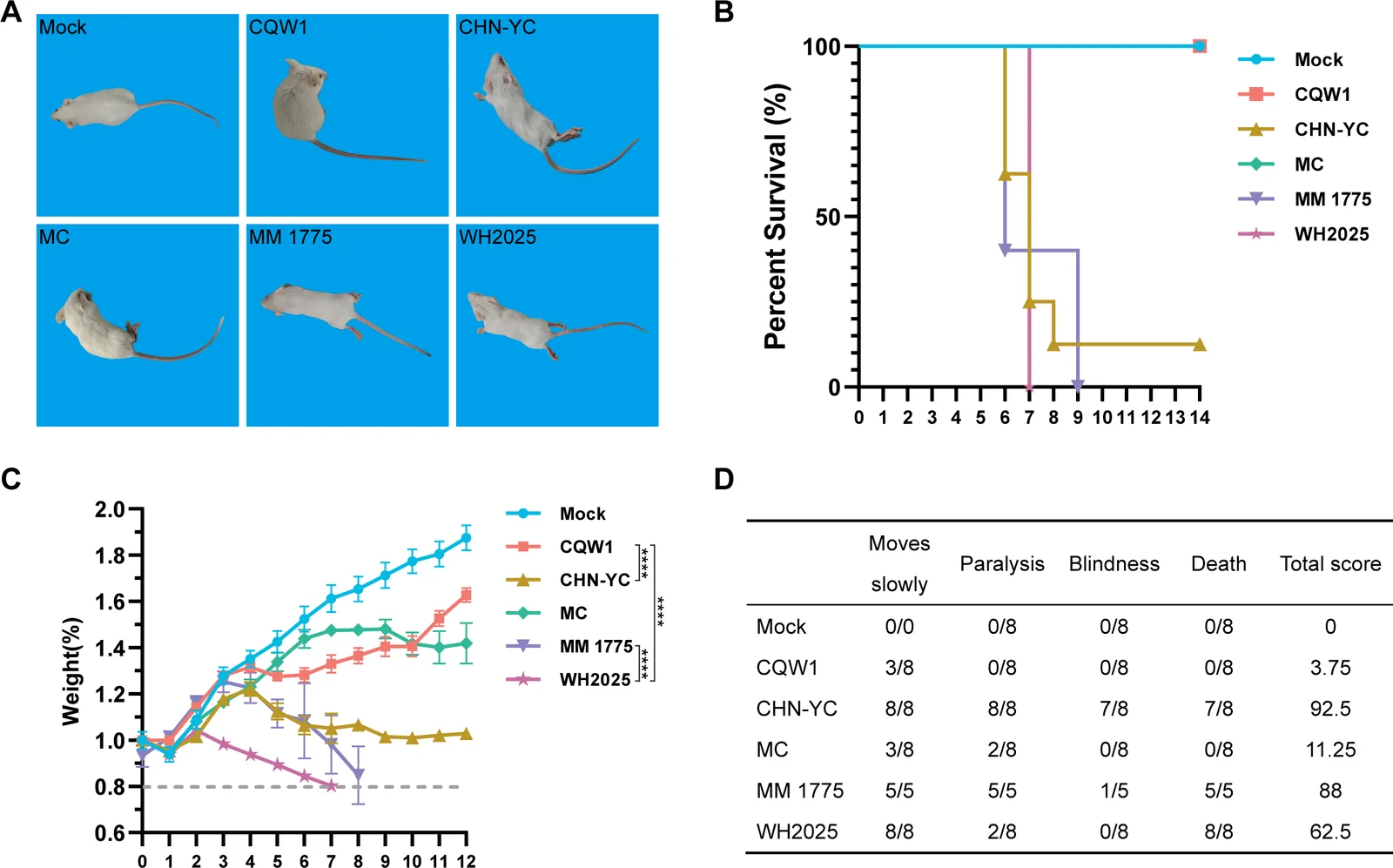
Pathogenicity of different TMUV isolates in mice. Three-week-old female Kunming mice were infected with TMUV via i.c. injection. Infected mice exhibited emaciation, limping, paralysis, and blindness (**A**). The percentage of survival (**B**) and body weight changes (**C**) were monitored until 14 days post-injection. Data are presented as means ± SD. The significance of the survival curve was determined by the Log-rank (Mantel-Cox) test. The significance of body weight was determined by two-way ANOVA. ****P < 0.0001. (**D**) Clinical symptom scores of mice infected with different TMUV isolates via i.c. injection. The total score was calculated as: 10% × item 1 + 30% × item 2 + 15% × item 3 + 45% × item 4.

Three-week-old female Kunming mice were infected with TMUV via i.c. injection. All isolates of TMUV are pathogenic to mice after inoculation, although to varying degrees. TMUV infection in mice leads to varying degrees of symptoms such as emaciation, slow movement or paralysis, and blindness (Fig. 4A). WH2025 and MM 1775 strains exhibited the highest pathogenicity in mice, causing 100% mortality; CHN-YC strain caused 87.5% mortality in mice; CQW1 and MC strains’ infections were not lethal to mice (Fig. 4B). All mice infected with TMUV exhibited a lower weight growth rate compared with the mock group (Fig. 4C). It is worth noting that the WH2025 strain caused reduced body weight at 3 dpi and continued to decrease until 7 dpi; the weight of mice infected with WH2025 strain was 20% lower than their original body weight, and all WH2025-infected mice were euthanized. The weight of mice infected with CHN-YC, MM 1775, or CQW1 strains began to reduce at 4 dpi; the weight loss of mice in the CHN-YC or MM 1775 strain infection group was significantly greater than that of mice infected with CQW1 or MC strain (Fig. 4C). The mice infected with the WH2025 strain began to exhibit clinical symptoms at 3 dpi, which included signs of mental depression, limb weakness, and reduced weight. CHN-YC or MM 1775 strain-infected mice exhibited clinical symptoms at 5 dpi and caused mental depression, paralysis, and blindness in mice. The MC or CQW1 strain-infected mice exhibited clinical symptoms after 8 dpi and led to slightly more disease compared to CHN-YC, MM 1775, or WH2025 strain infection (Fig. 4D). These results indicated that there were differences in pathogenicity among different TMUV isolates. CHN-YC, MM 1775, and WH2025 strains from clusters 2.1.2, 3.1, and the emerging cluster 3.2 showed strong mammalian pathogenicity.

### Pathological changes and viral loads in tissues

**Fig 5:**
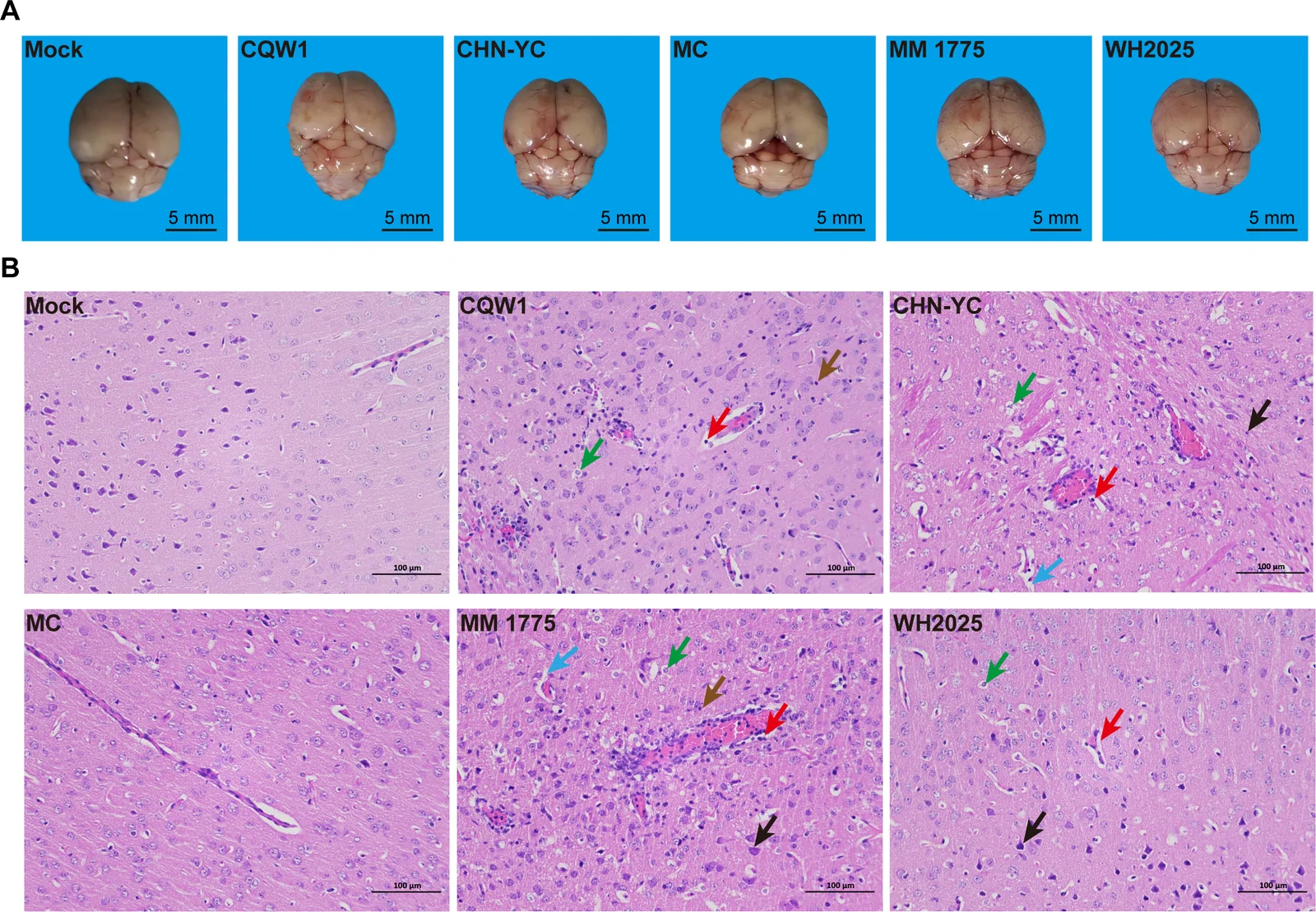
Pathological changes in the brain of TMUV-infected mice. (**A**) Gross lesions in the brain of infected mice at 5 days post-inoculation. The scale bar corresponds to 5 mm. (**B**) Histological changes in the cerebrum of infected mice at 5 days post-inoculation. Histopathological changes include neuronal shrinkage and hyperchromasia (black arrow), neuronal cytoplasmic rarefaction (green arrow), shrinkage and hyperchromasia of pyramidal cells and granular cells in the hippocampal region (purple arrow), perivascular edema (blue arrow), lymphocyte infiltration (red arrow), and gliosis (brown arrow). The scale bar corresponds to 100 μm.

**Fig 6:**
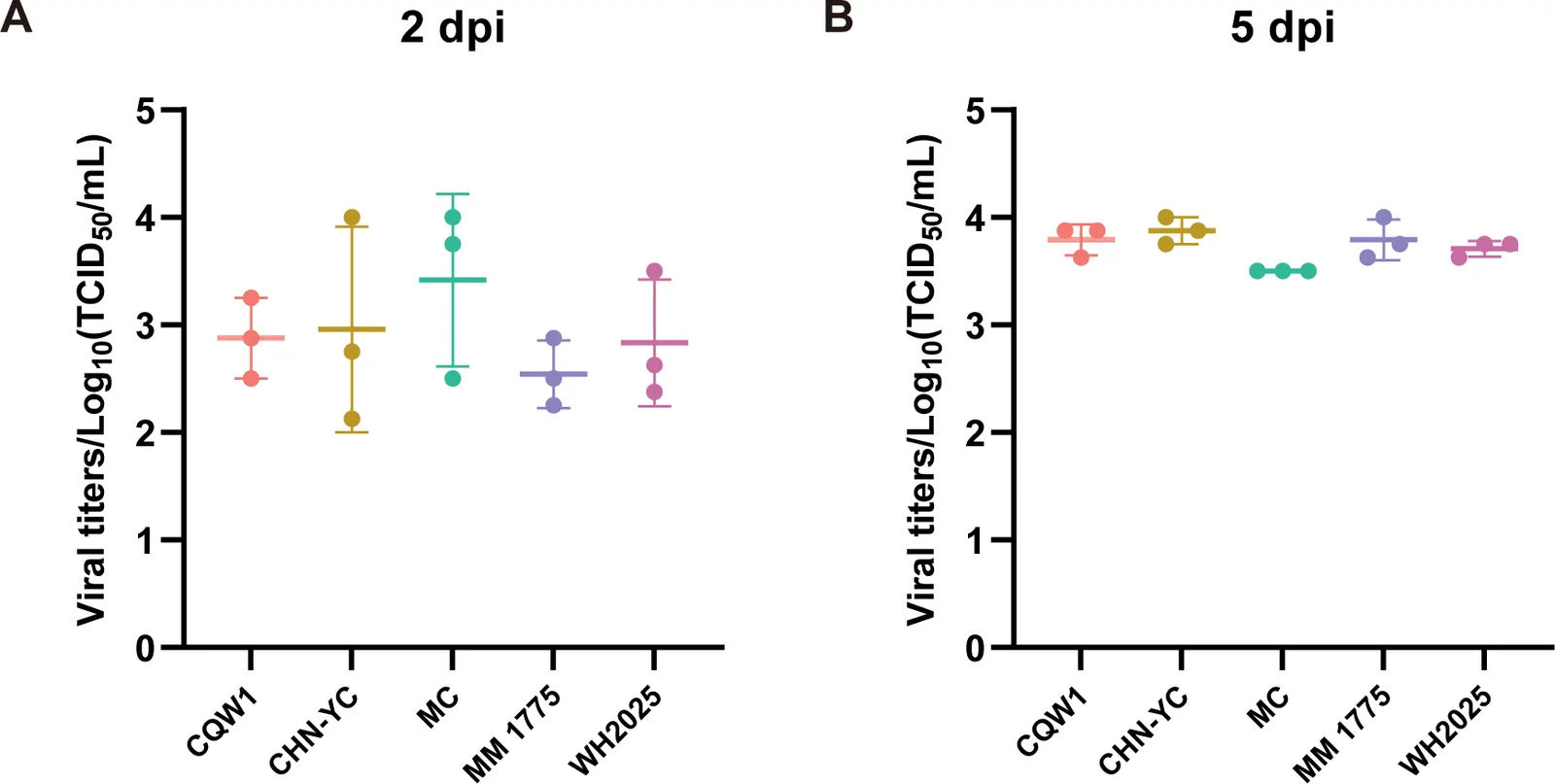
Viral loading in TMUV-infected mice. Viral loads in the brain tissue were titrated in BHK-21 cells at 2 dpi (**A**) and 5 dpi (**B**) post-injection of mice with TMUV.

At necropsy, hemorrhages were observed in the meninges of all TMUV-infected mice (Fig. 5A). In the brain tissue of mice infected with TMUV, pathological changes such as neuronal shrinkage, lymphocyte infiltration, and pyknosis and hyperchromasia of pyramidal cells and granular cells in the hippocampal region were observed (Fig. 5B). The viral titers in the brain were detected at 2 and 5 dpi, and viral copies were detected in other organs (heart, liver, spleen, lung, and kidney) at 5 dpi. Although infection with TMUV isolates from different clusters caused variable clinical symptoms, brain viral titers did not differ significantly at 2 dpi and 5 dpi (Fig. 6). Notably, the WH2025 strain induced marked neurological signs and weight loss beginning at 2 dpi, yet its viral load in the brain remained comparable to other isolates. To assess viral dissemination, visceral organs (heart, liver, spleen, lung, and kidney) were analyzed by RT-qPCR; however, no viral genome was detected in these tissues (data not shown). These results indicate that TMUV can successfully infect mice via intracranial injection, replicate in the brain, and induce corresponding neurological symptoms, but it cannot cross the blood-brain barrier to cause systemic infection.

### Cluster 3.1 TMUV triggers stronger innate immune and inflammatory responses

**Fig 7:**
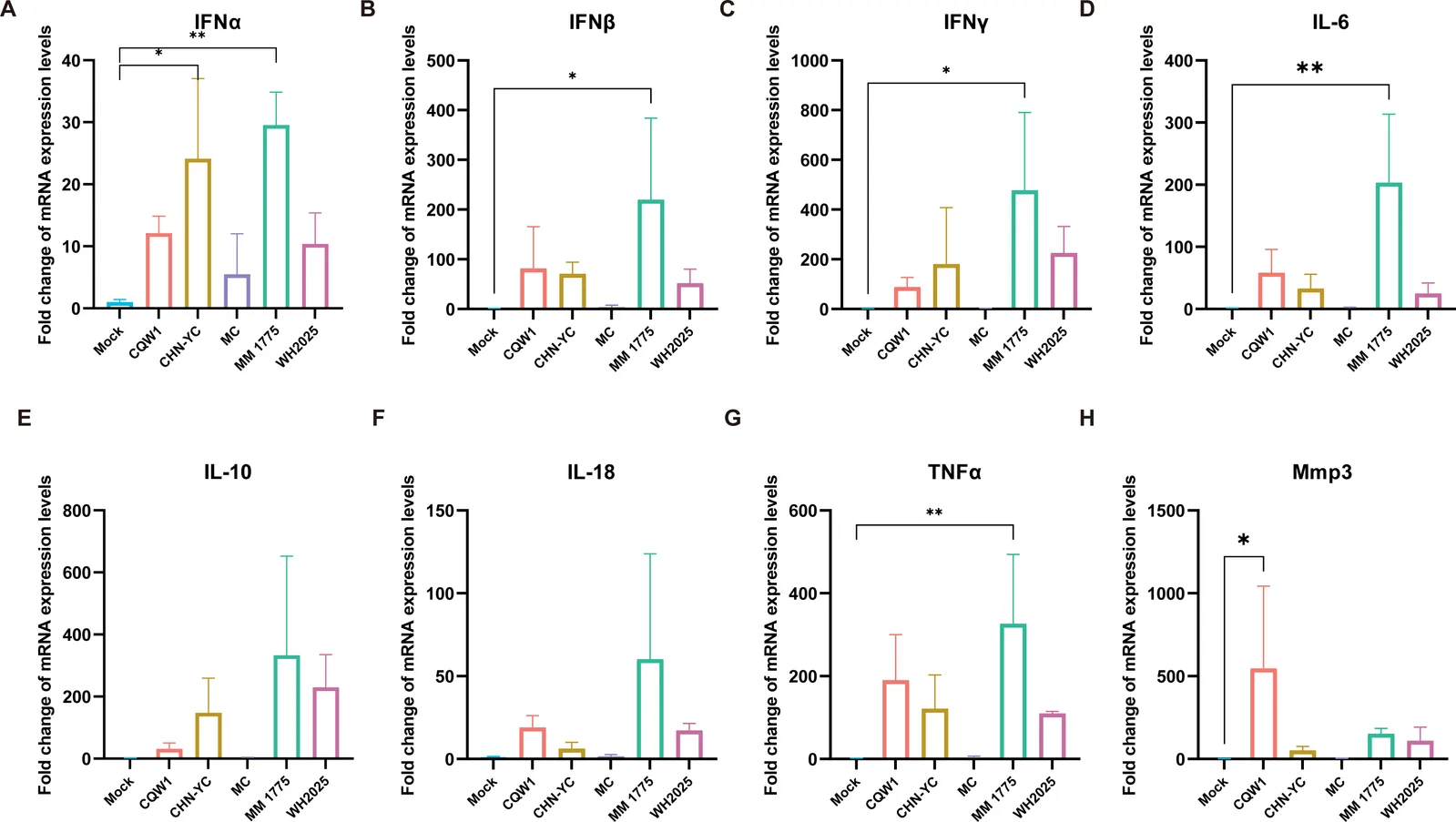
Immune and inflammatory responses in the brain of TMUV-infected mice. The transcription levels of IFNα (**A**), IFNβ (**B**), IFNγ (**C**), IL-6 (**D**), IL-10 (**E**), IL-18 (**F**), TNFα (**G**), and Mmp3 (**H**) in the brain of TMUV-infected mice (*n* = 3) were examined by RT-qPCR. The statistical significance was determined by the one-way ANOVA method. *P < 0.05 and **P < 0.01.

To evaluate strain-specific differences in host responses, mRNA expression of innate immune and inflammatory genes in mouse brains was quantified at 5 dpi (Fig. 7). The mosquito-origin MM 1775 strain elicited type I and II interferons at levels more than twice those induced by CQW1, CHN-YC, or WH2025 strains, whereas the MC strain caused only mild IFN-α upregulation. MM 1775 strain also drove an approximately 200-fold increase in IL-6 expression, though interindividual variation precluded statistical significance. Expression of matrix metalloproteinase 3 (Mmp3), a marker associated with blood–brain barrier disruption, was most strongly induced by CQW1, with CHN-YC, MM 1775, and WH2025 strains producing moderate increases. These results indicate that MM 1775 induces a more robust inflammatory response and innate immune response in infected mice, while CQW1, CHN-YC, and WH2025 induce comparable levels of inflammatory and innate immune responses, albeit lower than those induced by MM 1775.

## DISCUSSION

Tembusu virus has posed a significant economic threat to the poultry industry in China and Southeast Asia since its initial 2010 outbreak (1). Thus far, TMUV has only been reported in China and parts of Southeast Asia, likely reflecting a lack of systematic disease surveillance rather than its true geographic distribution. The discovery of a TMUV isolate genetically linked to Southeast Asian lineages in the droppings of long-distance migratory birds suggests that the virus’s endemic range is likely broader than currently recognized and may be undergoing continuous expansion (20). Beyond its agricultural impact, its placement within a genus that includes WNV and Usutu virus (known to cause fatal human encephalitis) raises immediate concerns regarding its zoonotic potential. Serological survey revealed a remarkably high prevalence of TMUV-specific antibodies not only among individuals in direct contact with infected duck populations but also in those with no known exposure, suggesting possible subclinical or environmental transmission. The recent confirmation of fatal TMUV infection in captive dolphins in Thailand, caused by a cluster 3.2 isolate (7), and the successful experimental infection of mice provide tangible evidence of its expanding host range into mammals.

Viral evolution under differing selection pressures has driven TMUV diversification into distinct phylogenetic clusters. Most isolates circulating among duck populations in mainland China belong to cluster 2 and were predominantly isolated before 2020, representing early epidemic lineages. In recent years, an increasing number of TMUV isolates belonging to cluster 3.2 have been detected in geese, chickens, quails, ducks, and mosquitoes (21, 22). Our analysis confirms the co-circulation of multiple lineages, with clusters 2.1.2 and 3 becoming predominant in recent years. Previous pathogenicity studies, often limited to a few isolates (13), could not provide a systematic, cross-cluster perspective. This fragmented approach has precluded a systematic understanding of how evolutionary divergence across the TMUV phylogeny translates to phenotypic differences in virulence and transmission.

In this study, we compared the *in vivo* and *in vitro* infection characteristics of major TMUV cluster isolates, including the prototype mosquito-derived isolate MM 1775, using both mice and *Culex quinquefasciatus* mosquitoes as model systems. Our parallel evaluation of key isolates reveals a clear virulence hierarchy in mammals. The emerging chicken-derived cluster 3.2 isolate (WH2025) and the duck-derived cluster 2.1.2 isolate (CHN-YC) exhibited significantly heightened pathogenicity compared to earlier cluster 2.1.1 and 2.2 isolates. Although different TMUV strains exhibit marked differences in neurovirulence in mice, their viral titers in the brain do not correlate with the clinical symptoms and neurovirulence they cause. This may be attributed to differences in the inflammatory response induced by different strains. However, in this study, due to the small number of samples tested and individual variations, no statistically significant differences were observed. Further studies are needed to explore the pathogenic mechanisms underlying the differential neurovirulence of various TMUV strains in mice. This indicates that contemporary TMUV evolution may favor genotypes with increased threat to mammalian hosts. However, due to the limited number of strains and the differences among different strains within the same cluster, the strains used in this study may not fully represent the pathogenic characteristics of TMUV strains within each cluster. Nevertheless, recent studies on the pathogenicity of TMUV cluster 2.1.2 and 3.2 isolates in mice have consistently shown that they possess strong neurovirulence (23, 24). However, the intracranial inoculation model used in this study, while effective for comparing neurovirulence among TMUV strains, bypasses natural infection routes and the blood–brain barrier. Thus, our results are primarily informative for neurovirulence assessment rather than for evaluating complete mammalian adaptation. To enhance the translational relevance of these findings, future investigations should include peripheral challenge models (e.g., intraperitoneal or subcutaneous inoculation).

The molecular basis for this enhanced virulence presents a compelling puzzle. We previously identified that E protein residue A487 significantly increased the pathogenicity of TMUV in mice, while E326 has been reported to be associated with low pathogenicity in mice (16, 25). In our previous study, we compared the amino acid sequences of all TMUV strains used in the phylogenetic analysis in this work and statistically identified recurrent amino acid mutations associated with different lineages (15). Intriguingly, the amino acid sequences of the E protein of TMUV strains within the same cluster are highly conserved, with regular mutations occurring between different clusters. In this study, except for the CQW1 strain, which contains the characteristic V487 mutation in its E protein, all other strains contain E326 and A487, yet these strains exhibit substantial differences in neurovirulence in mice. This paradox strongly implies that novel, unmapped genetic determinants in cluster 3.2 backgrounds compensate for or override the effects of individual residues. Identifying these compensatory mutations is a critical priority for future research.

In the mosquito vector, contemporary avian-derived isolates consistently outperformed the ancestral mosquito-derived MM 1775 isolate in replication and infection rates following both intrathoracic and oral infection. This indicates that epidemic TMUV isolates have evolved enhanced fitness in mosquitoes, potentially facilitating more efficient vector-borne transmission cycles. For the poultry industry, our findings highlight the need for cluster-aware surveillance. The differential virulence of cluster 3.2 in chickens and ducks (12) and its enhanced mammalian pathogenicity shown here suggest that this cluster may represent a particularly significant threat as it expands its host range. Understanding the phenotypic trade-offs associated with adaptation to different hosts (avians and mammals) and vectors is crucial for predicting outbreak dynamics and designing targeted control strategies.

In conclusion, our comprehensive evaluation of the major evolutionary clusters of the TMUV indicates that cluster 3.2 TMUV has become the currently dominant epidemic strain. Its pathogenic potential in mammals and its adaptability to vector carriers have significantly increased. The enhanced zoonotic and vector-borne transmission potential of TMUV warrants attention. The key sites and mechanisms responsible for this change in virological characteristics are crucial for the risk assessment and the development of prevention and control measures for TMUV strains and merit further in-depth investigation.

## Data Availability

The data that support the findings of this study are openly available in ScienceDB at https://doi.org/10.57760/sciencedb.31741.

## SUPPLEMENTAL MATERIAL

Table S1 spectrum.01094-26-s0001.xlsx Information on the TMUV isolates used in this study.

ASM does not own the copyrights to Supplemental Material that may be linked to, or accessed through, an article. The authors have granted ASM a non-exclusive, world-wide license to publish the Supplemental Material files. Please contact the corresponding author directly for reuse.
